# Supplementary material for: Electric control of spin transitions at the atomic scale
Source: Nat Commun. 2023 Oct 19;14:6612. doi: 10.1038/s41467-023-42287-2 (PMC10587172; doi:10.1038/s41467-023-42287-2)
Supplement: Supplementary file 1 — Supplementary Information [file 41467_2023_42287_MOESM1_ESM.pdf]

Supplementary Information for "Electric Control of Spin Transitions at the Atomic Scale"

Piotr Kot,[1] Maneesha Ismail,[1] Robert Drost,[1] Janis Siebrecht,[1] Haonan Huang,[1] and Christian R. Ast[1,*]

[1]*Max-Planck-Institut für Festkörperforschung, Heisenbergstraße 1, 70569 Stuttgart, Germany*

(Dated: September 29, 2023)

SUPPLEMENTARY METHODS

Magnetic Field/Bias Voltage Sweeps

We performed magnetic field/bias voltage sweeps on TiH molecules found on islands of MgO with a height of two monolayers (ML). Measurements were done by irradiating the junction at one frequency, and taking bias voltage dependent sweeps as a function of magnetic field. To minimize artifacts due to drift, we waited at least for two hours after approaching the tip and applying the microwave radiation prior to starting a sweep. To ensure that we do not drift off the molecular species under investigation, we performed atom tracking between bias sweeps while the magnetic field was ramping to the next value. In addition, we set the ramp rate of the magnet to relatively low values ($\approx$ 2.5 mT/s), ensuring minimal heating and slow adjustment of the STM junction. During each bias sweep, atom tracking was turned off and the tip position was set to hold. Lastly, we modulated the radiation at a chopping frequency of 107 Hz and set the demodulation frequency of our lock-in amplifier to the same frequency. This way we can pick up the ESR signal of the system in the lock-in amplifier and increase our signal to noise ratio [1]. These sweeps took anywhere from four to twelve hours depending on the number of points being measured.

SUPPLEMENTARY NOTE 1

Estimating the Electrically Induced Displacement

The estimate for the electrically induced displacement presented in the main text is based on considering the electric force acting in the junction, $F_{\mathrm{elec}} = eU/d$, and the opposing elastic force of the Ti-Mg bond, $F_{\mathrm{elas}} = -kz$. Here, U is the bias voltage in the junction, d is the tip sample distance, k is the elastic constant of the Ti-Mg bond and z is the Ti height relative to the Mg. We estimate the tip sample distance to be about 3 Å and we find a value for k in literature of 290 eV/nm^2 that is based on density functional theory and on a different adsorption site (for $\mathrm{TiH_O}$ instead of $\mathrm{TiH_{OO}}$) [2]. Using these values, we estimate the following displacement

$$\frac{z}{U} = -\frac{e}{kd} \approx -11.5\frac{\mathrm{fm}}{\mathrm{mV}}. \quad \text{(S1)}$$

This is to be understood as an estimate only, as we did not consider any screening or other effects.

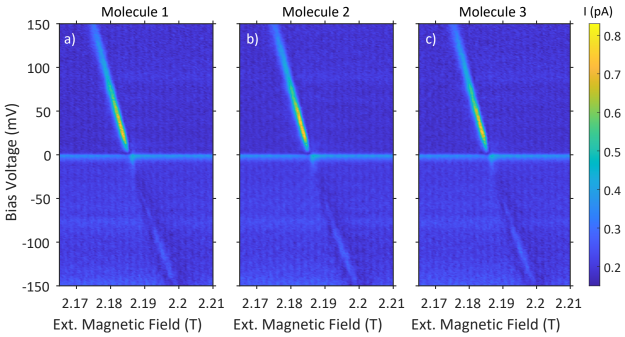

Supplementary Figure S1: **Bias dependent ESR-STM with varying microtips.** Magnetic field/bias voltage sweeps measured on three different $\mathrm{TiH_{OO}}$ molecules (U_{sp} = 100 mV, I_{sp} = 100 pA, f_{rf} = 61.545 GHz, U_{rf} = 20 mV). All three $\mathrm{TiH_{OO}}$ molecules show the same behavior.

SUPPLEMENTARY NOTE 2

Spin-Electric Coupling for Different Tips and $\mathrm{TiH_{OO}}$ Molecules

As a consistency check we performed magnetic field/bias voltage sweeps on various $\mathrm{TiH_{OO}}$ molecules found on the sample with the same tip. Fig. S1 shows three such sweeps on three different $\mathrm{TiH_{OO}}$ molecules from which we conclude that the measurements are consistent and reproducible.

Furthermore, the data presented in the main text was measured using two different ESR-functionalized tips. The first tip was used for the measurements shown in Fig. 1, Fig. 2 and Fig. 3(a). The second tip was used in the avoided crossing measurements shown in Fig. 3(d) and Fig. 4. In addition, over the course of this study we have observed spin-electric coupling in the ESR signal for five tips. Lastly, during our experiments we never encountered an ESR tip nor a TiH molecule that did not show spin-electric coupling.

SUPPLEMENTARY NOTE 3

Measurements on $\mathrm{TiH_O}$

Magnetic field/bias voltage sweeps were also performed on on-site TiH molecules ($\mathrm{TiH_O}$). Fig. S2(a) shows such a sweep where the ESR signal can be clearly seen. We see a linear shift of the ESR peak at positive bias voltages and no signal at negative bias voltages. We found that increasing the set point current of the magnetic field/bias voltage sweeps on $\mathrm{TiH_O}$ increased the linear shift of the

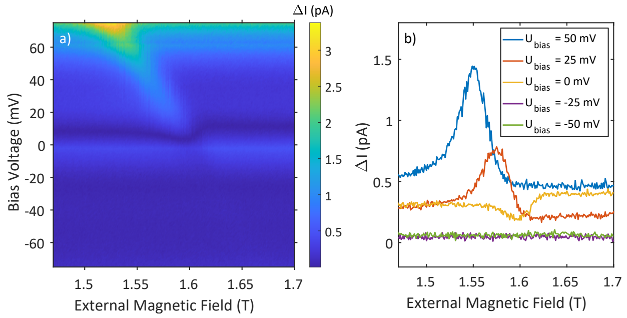

Supplementary Figure S2: **Bias dependent ESR-STM on TiH_O.** (a) Magnetic field/bias voltage sweep measured on a TiH_O molecule (U_{sp} = 100 mV, I_{sp} = 75 pA, f_{rf} = 19 GHz, U_{rf} = 20 mV). (b) ESR sweeps measured on TiH_O at different bias voltages. The evolution of the ESR peak shows a similar voltage dependence as for TiH_{OO}, but the spin-electric coupling is stronger.

ESR signal with respect to the bias voltage, which is consistent with our observations on TiH_{OO} molecules. We found that the shift of the TiH_O is much stronger than on the TiH_{OO} for similar setpoint currents. We attribute this to the tip being closer to the sample when measuring on TiH_O than when measuring on TiH_{OO}. This is due to the smaller local density of states on the TiH_O molecule, which leads to the tip sample distance being smaller on the TiH_O than on the TiH_{OO} for comparable set points. This is supported by the different appearance of the TiH_O compared to the TiH_{OO} as the TiH_{OO} molecules appear brighter than the TiH_O molecules (cf. Fig. 1(a) of the main text). Interestingly, for the TiH_O we were not able to measure at bias voltages above ≈ 80 mV, which may be due to orbital excitations inducing inelastic processes.

SUPPLEMENTARY NOTE 4

Extracting g-Factors and Tip Fields

To extract the bias voltage dependency of the g-factor and the magnetic field of the tip presented in the main text in Fig. 2, we measured magnetic field/bias voltage sweeps on a bridge site TiH molecule (TiH_{OO}) at four different microwave frequencies (i.e. Zeeman energies) and four different current set points. Fig. S3 shows magnetic field/bias voltage sweeps at four different current set points. We keep the x-axis scaling the same in all panels to more clearly show the effect of the tip-sample distance on the bias voltage shift of the ESR signal. Already there is a clear indication that the spin-electric coupling (SEC) is stronger at smaller tip-sample distances. Fig. S4 shows magnetic field/bias voltage sweeps measured at four different microwave frequencies. The horizontal features in all panels of Figs. S4 and S1 are due to the interaction of the microwaves with the background density of states and not related to the ESR signal (cf. [3, 4]).

We can extract the dependencies of the g-factor and the tip field on the bias voltage at a specific current set point by the procedure outlined in Fig. S5. We extract the magnetic field positions of the ESR signal maxima at each bias voltage from a magnetic field/bias voltage sweep and do a spline interpolation of the bias voltage vs. magnetic field points as shown in Fig. S5(a). In practice, bias voltages smaller than ±20 mV do not show a clear ESR signal, which we attribute to too low currents close to zero bias voltage. To bridge this gap, we interpolate the missing data points with a spline interpolation. We then use spline interpolations at four different microwave frequencies to find the positions of the ESR peaks on each magnetic field/bias voltage map. We then performed a linear fit at each bias voltage using Eq. (1) in the main text to extract the g-factor (slope) and B_{tip} (shift/offset). The spline interpolated data for each microwave frequency (i.e. Zeeman energy) at a current set point of 250 pA is plotted in Fig. S5(b) along with a linear fit at two bias voltages (±150 mV). Each linear fit yields the g-factor (slope) and the tip field B_{tip} (negative x-axis intercept) found at that bias voltage. By performing these linear fits over a continous range of bias voltages, we can plot the dependency of the g-factor and tip field B_{tip} with respect to the bias voltage that we have presented in Fig. 2 of the main text.

To demonstrate the overall consistency of this multidimensional fit, we plot the extracted ESR peak positions from the data using spline interpolations along with the ESR resonance positions calculated using the g-factor and B_{tip} values found with our linear fits. This comparison is shown in Fig. S6 for all microwave frequencies and current setpoints. We see an overall good agreement and a continuous evolution. To illustrate the agreement quantitatively, we calculate the difference between the experimental data and the modeled resonances, which is shown in Fig. S7 for the corresponding data in Fig. S6. We see that the deviations are generally small and never exceeding 2 mT. Therefore, we conclude that we have an overall consistent model.

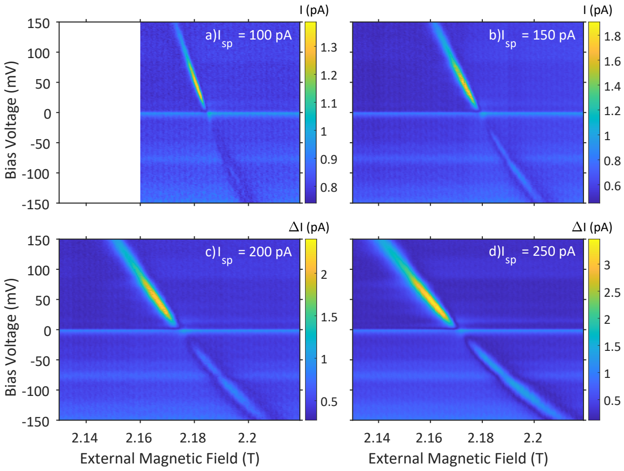

Supplementary Figure S3: **Bias dependent ESR-STM at varying set points.** Magnetic field/bias voltage sweeps measured at different tip-sample distances (U_{sp} = 100 mV, f_{rf} = 61.545 GHz, U_{rf} = 20 mV, (a) I_{sp} = 100 pA, (b) I_{sp} = 150 pA, (c) I_{sp} = 200 pA, (d) I_{sp} = 250 pA)

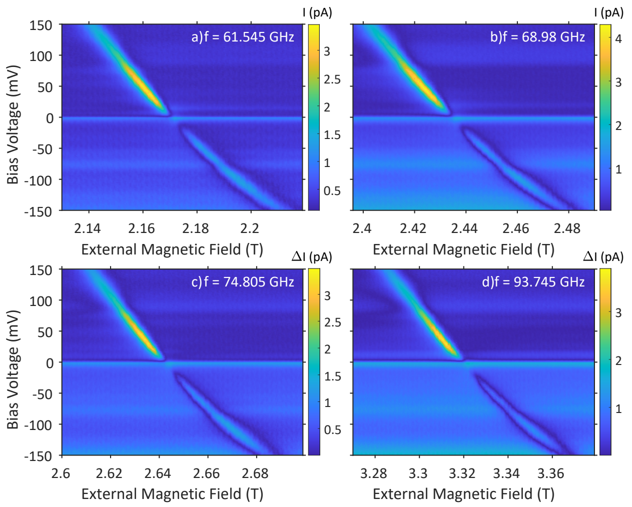

Supplementary Figure S4: **Bias dependent ESR-STM at varying frequencies.** Magnetic field/bias voltage sweeps measured at different microwave frequencies (U_{sp} = 100 mV, I_{sp} = 250 pA, U_{rf} = 20 mV, (a) f_{rf} = 61.545 GHz, (b) f_{rf} = 68.98 GHz, (c) f_{rf} = 74.805 GHz, (d) f_{rf} = 93.745 GHz)

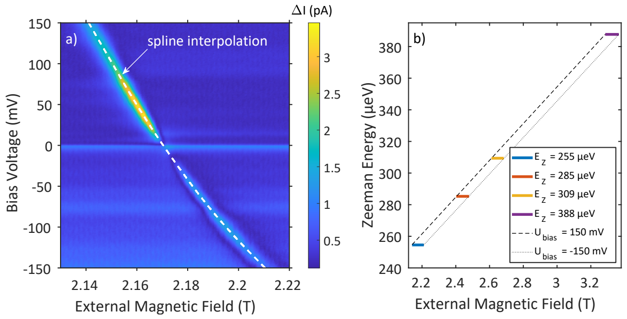

Supplementary Figure S5: **g-factor and tip field extraction.** (a) Magnetic field/bias voltage sweep with a dashed line indicating the spline interpolation

performed over the full bias voltage range (U_{sp} = 100 mV, f_{rf} = 61.545 GHz, U_{rf} = 20 mV and I_{sp} = 250 pA). (b) Representation of the linear fits at each bias voltage to extract the bias voltage dependencies of the g-factor and tip field B_{tip}. Two linear fits are shown at 150 mV and −150 mV represented by the dashed and dotted lines, respectively I_{sp} = 250 pA.

We also calculate the effective frequency shifts at 250 pA that result from the bias dependencies of the g-factor and tip field B_{tip}. This is done by fitting a line through the positive bias parts of the data presented in Fig. 2(c) and (d) of the main text, and then inserting each of these fits into the ESR equation (cf. main text)

$$E_Z = hf_{res} = g\mu_B(B_{ext} + B_{tip}). \tag{S2}$$

For the frequency shift due to the g-factor bias voltage dependence, we keep the tip field B_{tip} constant and vice versa. The choice of the constant value changes the value of the frequency dependencies only slightly. In our calculations we took these constant values to be the value of the g-factor or tip field B_{tip} at zero bias, respectively.

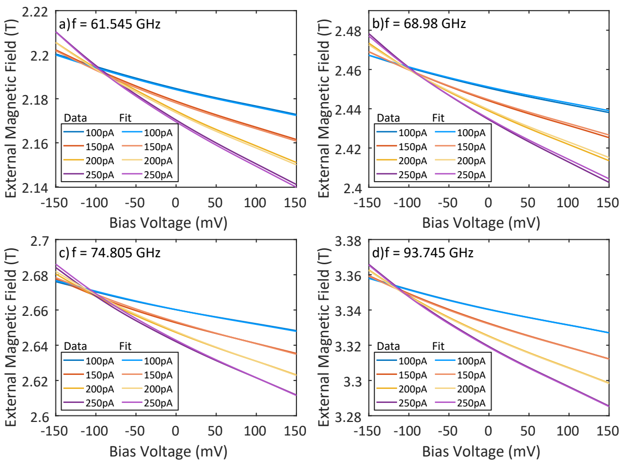

Supplementary Figure S6: **g-factor and tip field consistency check.** Comparison of the extracted ESR peak positions with the peak positions calculated from the fitted g-factors and tip fields B_{tip}. The different panels show the different microwave frequencies. We find generally good agreement for all frequencies and current setpoints.

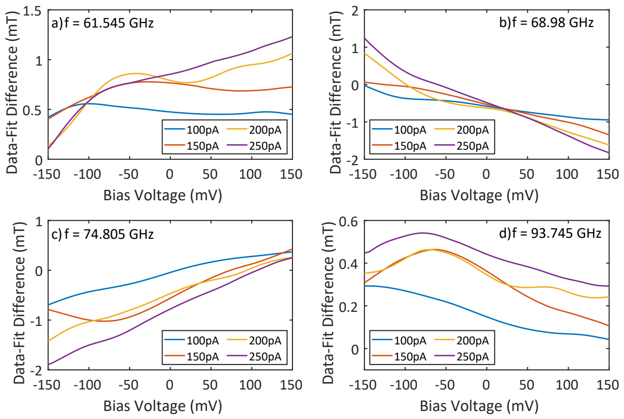

Supplementary Figure S7: **g-factor and tip field data and reproduction comparison.** Differences between the extracted and the calculated ESR peak positions shown in Fig. S6. The deviations are always less than 2 mT indicating good overall agreement.

SUPPLEMENTARY NOTE 5

Tip Approach

The coupling of the TiH molecule to the substrate can be inferred from the evolution of the tunnel junction transmission as a function of the tip sample distance. A similar situation has been analyzed previously in a different context [5]. Assuming that the TiH molecule is coupled to the substrate by the molecule-substrate coupling Γ_s and to the tip by the molecule-tip coupling Γ_t, the junction transmission τ can be written as [5, 6]

$$\tau = \frac{4\Gamma_s\Gamma_t}{(\Gamma_s + \Gamma_t)^2} \overset{\Gamma_t \ll \Gamma_s}{=} \frac{4\Gamma_t}{\Gamma_s}. \tag{S3}$$

The transmission τ describes the junction conductance in units of the quantum of conductance $G_0 = 2e^2/h$, where

e is the electron charge and h is Planck's constant. Since our junction is in the tunneling limit, i.e. $\Gamma_t \ll \Gamma_s$, we can easily see that a change in the molecule-substrate coupling Γ_s has a direct impact on the evolution of the junction transmission. We can reasonably assume that in the tunneling regime, the molecule-tip coupling Γ_t increases exponentially with decreasing tip-sample distance. If the molecule-substrate coupling Γ_s increases/decreases as the tip-sample distance decreases, the junction transmission τ will evolve less/more than exponentially, respectively. The tip approach for the tunnel junction measured in the main text in Fig. 2 is shown in Fig. S8(a). The blue line represents the data, while the red line represents an exponential fit to the data points at z-positions > 60 pm. A small but clear subexponential deviation of the data can be seen. The relative difference between data and fit is also plotted in Fig. S7(b) indicating that the junction transmission evolves below the fitted exponential evolution. From this behavior, we conclude that the molecule-substrate coupling Γ_s increases as the tip approaches the molecule. Therefore, it is likely that the molecule is pushed towards the surface in this approach range. This provides an overall consistent picture of an increasing g-factor as the molecule-substrate distance decreases [7] and explains the evolution of the g-factor at zero bias voltage for decreasing tip-sample distance.

SUPPLEMENTARY NOTE 6

Modelling Coupled Spins

The models presented in Fig. 3(b) and (c) of the main text are based on a coupled spin Hamiltonian found in literature [8–10]:

$$H = -\mu_B(B_{ext} + B_{tip})g_1\hat{S}_1^z - \mu_B B_{ext} g_2 \hat{S}_2^z + J\hat{S}_1 \cdot \hat{S}_2 + D(3\hat{S}_1^z\hat{S}_2^z - \hat{S}_1 \cdot \hat{S}_2). \quad \text{(S4)}$$

This Hamiltonian works on the spin operators of the coupled spins, where g_1 and g_2 are the g-factors of the TiH molecule beneath the tip and beside the tip, respectively, B_{tip} is the tip field that is only considered to affect the TiH molecule beneath the tip, B_{ext} is the external magnetic field, J is the Heisenberg interaction energy between the two spins, and D is the dipole interaction between the two spins. For the dipole interaction, we estimate $D = 13.3$ MHz for the more distant dimer and $D = 50$ MHz for the closer dimer, which is a significantly smaller contribution than the other interactions.

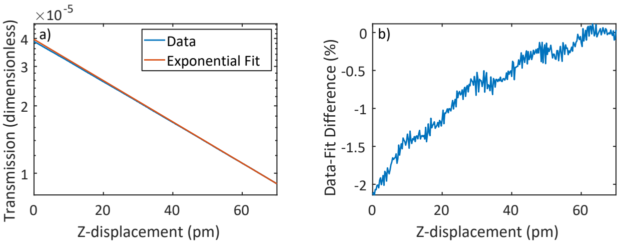

Supplementary Figure S8: **Junction transmission analysis.** (a) Junction transmission as a function of z-displacement (tip-sample distance). The exponential fit is done in the low transmission regime revealing the sub-exponential evolution of the data. (b) Difference between data and fit normalized to the fit showing the sub-exponential evolution of the tip-approach.

	Avoided Crossing		Large J	
I_{sp}	400 pA	400 pA	1 nA	1 nA
U_{sp}	100 mV	100 mV	150 mV	150 mV
U_{bias}	0 mV	200 mV	0 mV	200 mV
J	0.669 GHz	0.669 GHz	61.11645 GHz	61.11645 GHz
D	13.3 MHz	13.3 MHz	50 MHz	50 MHz
g_1	1.92	1.925	2.0703	2.103
g_2	1.975	1.973	1.87	1.913
B_{tip}	0 mT	14 mT	20 mT	36 mT

Table SI: Fit parameters for the two dimers presented in the main text. The bias voltage values in the table are the extremal values at the edge of the interval. The parameters for the bias voltage values in between are linearly interpolated. The corresponding g-factors and tip fields differ between the dimers because they were measured with different tips and at different current and voltage set points I_{sp} and U_{sp}.

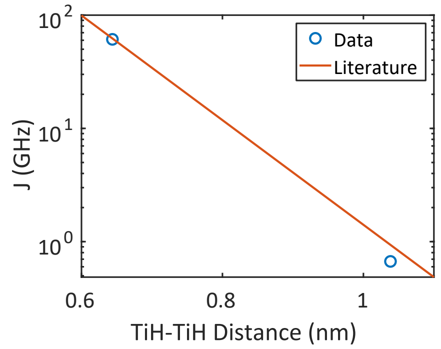

Supplementary Figure S9: **Exchange coupling comparison.** Comparison of the fit parameters for the exchange coupling J in the two dimers with the exponential dependence reported in literature [9].

Modelling of the experimentally observed transitions is done by considering the energy difference between two eigenvalues of the spin Hamiltonian. Modeling the TiH molecules as spin-½ systems, the spin Hamiltonian in Eq. (S4) can be diagonalized to analytically find the four eigenstates, three triplet states ($|T_+\rangle$, $|T_0\rangle$ and $|T_-\rangle$) and one singlet state ($|S\rangle$). In the case of the avoided crossing, we observe four transitions with energies: $|E_{T_0} - E_{T_-}|$, $|E_{T_0} - E_{T_+}|$, $|E_S - E_{T_-}|$, and $|E_S - E_{T_+}|$ [8?]. We then equate these energy differences to the energy hf of the microwave radiation, which leads to the following set of equations:

$$hf = |E_{T_O} - E_{T_+}| = \frac{1}{2}[J + 2D + \mu_B(g_1(B_{ext} + B_{tip}) + g_2 B_{ext}) - \sqrt{(J-D)^2 + (\mu_B(g_1(B_{ext} + B_{tip}) + g_2 B_{ext}))^2}], \quad \text{(S5)}$$

$$hf = |E_{T_O} - E_{T_-}| = \frac{1}{2}[J + 2D - \mu_B(g_1(B_{ext} + B_{tip}) + g_2 B_{ext}) - \sqrt{(J-D)^2 + (\mu_B(g_1(B_{ext} + B_{tip}) + g_2 B_{ext}))^2}], \quad (S6)$$

$$hf = |E_S - E_{T_+}| = \frac{1}{2}[J + 2D + \mu_B(g_1(B_{ext} + B_{tip}) + g_2 B_{ext}) + \sqrt{(J-D)^2 + (\mu_B(g_1(B_{ext} + B_{tip}) + g_2 B_{ext}))^2}], \quad (S7)$$

$$hf = |E_S - E_{T_-}| = \frac{1}{2}[J + 2D - \mu_B(g_1(B_{ext} + B_{tip}) + g_2 B_{ext}) + \sqrt{(J-D)^2 + (\mu_B(g_1(B_{ext} + B_{tip}) + g_2 B_{ext}))^2}]. \quad (S8)$$

Using this set of equations we can solve for four different external magnetic fields B_{ext} ($B_{T_0T_+}$, $B_{T_0T_+}$, B_{ST_+} and B_{ST_-}) numerically using input values for J, D, g_1, g_2 and B_{tip}. The microwave frequency f is a known input quantity. The resulting external magnetic field values B_{ext} represent the positions of the ESR peaks on the external magnetic field axis for the given transitions. In the case of the dimer with the stronger interaction energy, we model the positions of the ESR peaks by considering the following transition energies: $|E_{T_0} - E_{T_-}|$, $|E_{T_0} - E_{T_+}|$ and $|E_S - E_{T_0}|$ [9].

To incorporate the effect of the bias voltage in the modelling we assume a linearly dependence of g_1, g_2, and B_{tip} on the bias voltage in the range from 0 mV to 200 mV. This is based on the results presented in Fig. 2(c) and (d) of the main text. We find that to get accurate results, g_2 also has to shift with the bias voltage, which implies that the electric field of the tip still affects the TiH molecule next to the tip apex. This is to be expected as the tip and sample can be approximated as a plate capacitor close to the tip apex. Furthermore, we assume that J and D are not affected by the bias voltage. We choose the values for J according to the exponential distance dependence between the molecules in the dimer that has been established previously [9–11]. The comparison is shown in Fig. S9, where the red line is given by $J = J_0 \exp(-(r - r_0)/d)$ with $r_0 = 0.72$ nm, $d = 94$ pm, and $J_0 = 27.7$ GHz [11]. We then input a constant J and D into our set of equations and solve for the external magnetic field values B_{ext} constituting the positions of the ESR peaks as described above. For each set of g_1, g_2 and B_{tip}, we solve for $B_{T_0T_+}$, $B_{T_0T_+}$, B_{ST_+} and B_{ST_0} for the case of the dimer with the avoided crossing, and $B_{T_0T_+}$, $B_{T_0T_+}$ and B_{ST_0} for the case of the dimer with a larger interaction energy. Finally, we superimpose the calculated ESR transitions over the data to find the parameters with the best fit. The fit parameters g_1, g_2, B_{tip}, D and J for the two different dimers are presented in Table SI.

To plot the modelled eigenergies in Fig. 3(b) and (c) of the main text, we simply input our estimated values for J and linearly changing g_1, g_2 and B_{tip} into the diagonalized eigenergies of Eq. (S4). Here, the x-axis in Fig. 3(b) and (c) is an "effective" bias voltage that we model with linearly shifting values for g_1, g_2 and B_{tip}, but for a constant external magnetic field B_{ext}. Therefore, the evolution of the energy levels in Fig. 3(b) and (c) and the experimental data in Fig. 3(a) and (d) are not directly comparable.

SUPPLEMENTARY REFERENCES

* Corresponding author; electronic address: c.ast@fkf.mpg.de

[1] T. S. Seifert, S. Kovarik, C. Nistor, L. Persichetti, S. Stepanow, and P. Gambardella, *Single-atom electron paramagnetic resonance in a scanning tunneling microscope driven by a radio-frequency antenna at 4 K,* Physical Review Research **2**, 013032 (2020).

[2] A. Ferrón, S. A. Rodríguez, S. S. Gómez, J. L. Lado, and J. Fernández-Rossier, *Single spin resonance driven by electric modulation of the g-factor anisotropy,* Physical Review Research **1**, 033185 (2019).

[3] P. K. Tien and J. P. Gordon, *Multiphoton Process Observed in the Interaction of Microwave Fields with the Tunneling between Superconductor Films,* Physical Review **129**, 647 (1963).

[4] P. Kot, R. Drost, M. Uhl, J. Ankerhold, J. C. Cuevas, and C. R. Ast, *Microwave-assisted tunneling and interference effects in superconducting junctions under fast driving signals,* Physical Review B **101**, 134507 (2020).

[5] H. Huang, R. Drost, J. Senkpiel, C. Padurariu, B. Kubala, A. L. Yeyati, J. C. Cuevas, J. Ankerhold, K. Kern, and C. R. Ast, *Quantum phase transitions and the role of impurity-substrate hybridization in Yu-Shiba-Rusinov states,* Communications Physics **3**, 199 (2020).

[6] J. C. Cuevas and E. Scheer, *Molecular Electronics: An introduction to Theory and Experiment*, 2nd ed. (World Scientific, Singapore, 2017).

[7] M. Steinbrecher, W. M. J. van Weerdenburg, E. F. Walraven, N. P. E. van Mullekom, J. W. Gerritsen, F. D. Natterer, D. I. Badrtdinov, A. N. Rudenko, V. V. Mazurenko, M. I. Katsnelson, A. van der Avoird, G. C. Groenenboom, and A. A. Khajetoorians, *Quantifying the interplay between fine structure and geometry of an individual molecule on a surface,* Physical Review B **103**, 155405 (2021).

[8] L. M. Veldman, L. Farinacci, R. Rejali, R. Broekhoven, J. Gobeil, D. Coffey, M. Ternes, and A. F. Otte, *Free coherent evolution of a coupled atomic spin system initialized by electron scattering,* Science **372**, 964 (2021).

[9] Y. Bae, K. Yang, P. Willke, T. Choi, A. J. Heinrich, and C. P. Lutz, *Enhanced quantum coherence in exchange coupled spins via singlet-triplet transitions,* Science Advances **4**, eaau4159 (2018).

[10] K. Yang, Y. Bae, W. Paul, F. D. Natterer, P. Willke, J. L. Lado, A. Ferrón, T. Choi, J. Fernández-Rossier, A. J. Heinrich, and C. P. Lutz, *Engineering the Eigenstates of Coupled Spin-1/2 Atoms on a Surface,* Physical Review Letters **119**, 227206 (2017).

[11] K. Yang, S.-H. Phark, Y. Bae, T. Esat, P. Willke, A. Ardavan, A. J. Heinrich, and C. P. Lutz, *Probing resonating valence bond states in artificial quantum magnets,* Nature Communications **12**, 993 (2021).
